# Supplementary material for: Analyzing the ER stress response in ALS patient derived motor neurons identifies druggable neuroprotective targets
Source: Front Cell Neurosci. 2024 Jan 19;17:1327361. doi: 10.3389/fncel.2023.1327361 (PMC10834640; doi:10.3389/fncel.2023.1327361)
Supplement: Supplementary file 5 [file Data_Sheet_1.docx]

Supplementary Material

Analyzing the ER stress response in ALS patient derived motor neurons identifies druggable neuroprotective targets

Michelle E. Watts, Richard M. Giadone, Alban Ordureau, Kristina M. Holton, J. Wade Harper, Lee L. Rubin^*^

*** Correspondence:** Dr. Lee L. Rubin: [lee_rubin@harvard.edu](mailto:lee_rubin@harvard.edu)

**Supplementary Figure S1. Extended analyses of iPSC-derived MN cultures and response to proteostatic stressors.** (A) MN and non-MN viability from 1016 healthy control cultures at low density (25K/96 well or 12.5K/96 well) or with maturation (14 days in vitro (DIV) at 50K/96well) after 48hrs exposure to increasing doses of ER stressors. Nb=3, nt=2, two-way ANOVA; 25K thapsigargin- p = 7.67x10^-6^, 12.5K thapsigargin- p = 0.000258, 2week thapsigargin- p = 0.000691, 25K tunicamycin- p = 0.008. 12.5K tunicamycin- p = 2.63x10^-7^, 2week tunicamycin- p = 0.002. For simplicity, stars indicating significance are shown for the condition with the least significance. (B) Quantification of MN and non-MN viability 48hrs after treatment with increasing concentrations of MG132 (1016 healthy control line). Nb=3, nt=12, two-way ANOVA, p = 0.394. (C) Quantification of MN and non-MN viability after treatment with 1μM MG132 for various lengths of time (1016 healthy control line). (D) MN and non-MN viability from low density cultures (25K/96 well or 12.5K/96 well) or mature cultures (14 days in vitro (DIV) at 50K/96well) after 48hrs exposure to increasing doses of MG132 (1016 healthy control line). Nb=3, nt=2, two-way ANOVA; 25K- p = 0.894. 12.5K- p = 0.947. 2week- p = 0.284. (E) Western blot quantification of phosphorylated eIF2α with increasing time of DMSO, 1μM thapsigargin or 1μM tunicamycin (1016 healthy control line); Nb = 3, Nt=1, two tailed students t.test to DMSO control p<0.05 = *, p<0.01 = **. (F) Quantification of spliced XBP1 template, normalized to unspliced XBP1 template after 2 and 4hrs of DMSO, 1μM thapsigargin or 1μM tunicamycin treatment (1016 healthy control line); Nb = 3, Nt=1, two tailed students t.test to DMSO ctrl p<0.05 = *, p<0.01 = **. (G) Western blot quantification of BiP at 8 and 24hrs of DMSO, 1μM thapsigargin or 1μM tunicamycin treatment (1016 healthy control line); Nb = 3, Nt=1, two tailed students t.test to DMSO control p<0.05 = *, p<0.01 = **. (H) Western blot quantification of CHOP at 8 and 24hrs of DMSO, 1μM thapsigargin or 1μM tunicamycin treatment (1016 healthy control line); Nb = 3, Nt=1, two tailed students t.test to DMSO control p<0.05 = *, p<0.01 = **. (I) Western blot quantification of cleaved caspase 3 at 8 and 24hrs of DMSO, 1μM thapsigargin or 1μM tunicamycin treatment (1016 healthy control line); Nb = 3, Nt=1, two tailed students t.test to DMSO control p<0.05 = *, p<0.01 = **. (J) Healthy control 1016A, SOD1, and TDP43 iPSCs were differentiated into MN cultures (containing MN and non-MN cell populations) and exposed to 1 μM thapsigargin for 16 hours. qRT-PCR revealed no differences in response to thapsigargin exposure across healthy control and ALS MNs by expression of UPR-associated genes ATF6, IRE1, CHOP, and BiP. Nb = 2, Nt = 3. Biological replicate experiments denoted as Nb, each with technical replicate experiments nt. Data are mean value +/- SEM. p<0.05 was considered statistically significant and denoted in graphs with a *, p<0.01 **, p<0.001 ***, and P<0.0001 ****.

**Supplementary Figure S2. Overview of global phosphoproteomics experiment.** (A) Schematic of the ER stress and protection assay and the subsequent quantitative proteomics analysis pipeline. (B) Individual, separated proteomic volcano plots for all treatment conditions. -Log_10_(p-value) is graphed on the Y-axis, Log_2_(fold change) graphed on the X-axis for all plots. (C) Individual, separated phosphoproteomic volcano plots for all treatment conditions. -Log_10_(p-value) is graphed on the Y-axis, Log_2_(fold change) graphed on the X-axis for all plots.

**Supplementary Figure S3. Approach to identifying viable MNs.** (A) Example nuclear size exclusion parameters and Hoechst intensity thresholding used to identify the viable cell population. Histograms to right of selection script and input image demonstrate 2 distinct cell populations, live or dead, with live cells demonstrating a nuclear area >~37-55μm2 and Hoechst intensities lower than the threshold brightness of pyknotic nuclei (18,000 in this example). (B) Viable cell script accuracy confirmed with LIVE/DEAD Viability/Cytotoxicity Kit, for mammalian cells (Life Technologies L3224). (C) Example Isl1/2 intensity thresholding used to identify the viable MN population. Histogram to right of selection script and input image demonstrate that selected Isl1/2+ cell populations must have an Isl1/2 intensity greater than the basal intensity (>8000 in this example).

**Supplementary Figure S4. Neurite tracing of β-Tubulin III staining**. Representative image analysis pipeline to track neurites using B-Tubulin III staining.

**Supplementary Data Set 1. ER stress and protection proteomics and phosphoproteomics dataset.** Quantified proteins and phosphoproteins are displayed with corresponding log_2_foldchange with each treatment, compared to indicated control.

**Supplementary Video Files**. Automated live cell imaging of 1016A healthy control iPSC-derived MN cultures treated with DMSO (1), 1µM thapsigargin (2), or 1µM tunicamycin (3). Images were taken every 6hrs for 48hrs.

**Supplementary Table S1. Overview of quantified phosphopeptides and peptides**

| **Data Set** | **# Peptides (Set 1)** | **# Peptides (Set 2)** | **# Unique Proteins** | **# Unique Phosphorylation Sites** |
| --- | --- | --- | --- | --- |
| Stress+Protection (Protein) | 67,357 | 59,708 | 6,697 |  |
| Stress+Protection (Phospho-protein) | 28,399 | 28,973 | 2,764 | 7,999 |

**Supplementary Table S2. Protective processes implicated by proteomic analyses.**

| **Protective Compound** | **Implicated Protective Process** | **Specific Implicated Targets** |
| --- | --- | --- |
| Kenpaullone | Microtubule Dynamics | Phospho-CLASP2, Phospho-DPYSL2, MAP1B, MAPT, TPP, Kif11 |
| Kenpaullone + MAP4K4i | Signaling | GSK3β, PKC, JNK, c-JUN, mTOR/S6K, RAF, PIM |
| Kenpaullone + MAP4K4i | Metabolism | ACSL4, HMG-CoA Reductase, SCD, ODC, CHDH |
|  | (Fatty Acid, Lipid, Cholesterol) |  |
| Kenpaullone + MAP4K4i | Receptors | Insulin, GABA |
| Kenpaullone + MAP4K4i | Cell Cycle Proteins | Cdc42, CDK3/5 |
| Kenpaullone + MAP4K4i | Calcium Dynamics | Cam2K, CSNK2 |
| Kenpaullone + MAP4K4i | ER-Golgi Anterograde Transport | GBF1, Phospho-EXOC1 |
| MAP4K4i | HDACs | HDAC1, 2, 5 |
