# Supplementary figures and images for: Analyzing the ER stress response in ALS patient derived motor neurons identifies druggable neuroprotective targets
Source: Front Cell Neurosci. 2024 Jan 19;17:1327361. doi: 10.3389/fncel.2023.1327361 (PMC10834640; doi:10.3389/fncel.2023.1327361)

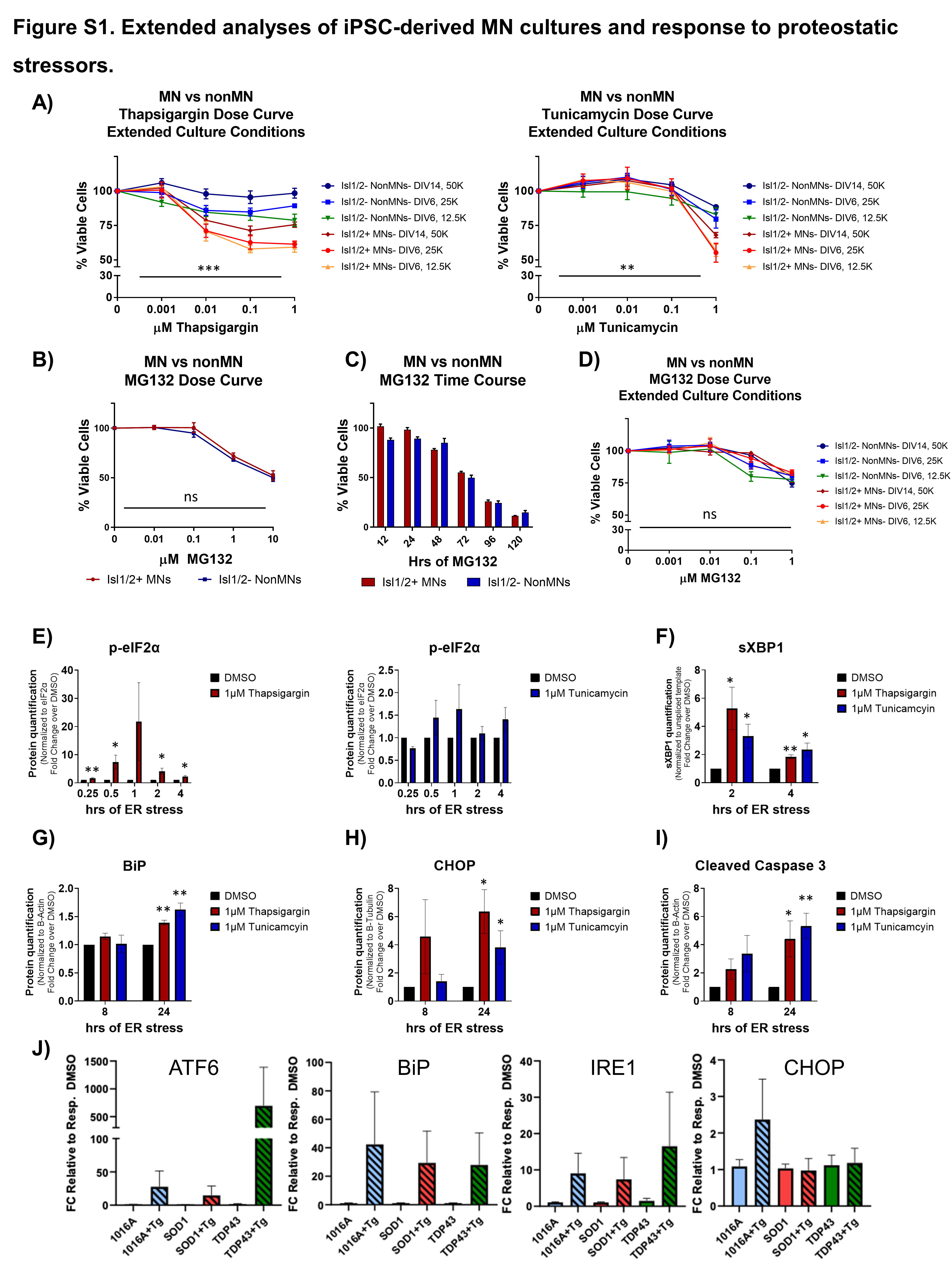

Supplement: Supplementary file 6 [file Image_1.TIF]

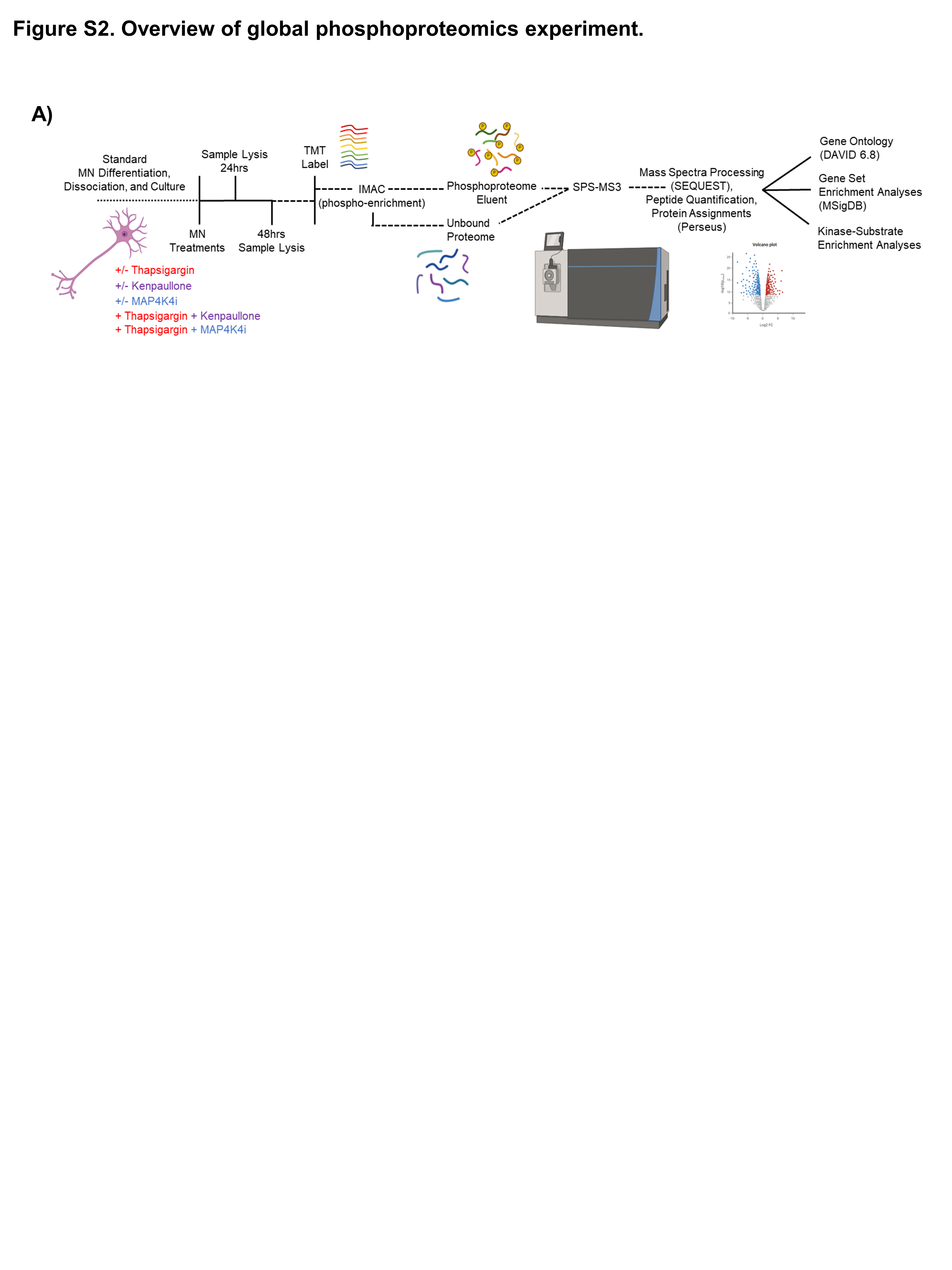

Supplement: Supplementary file 7 [file Image_2.TIF]

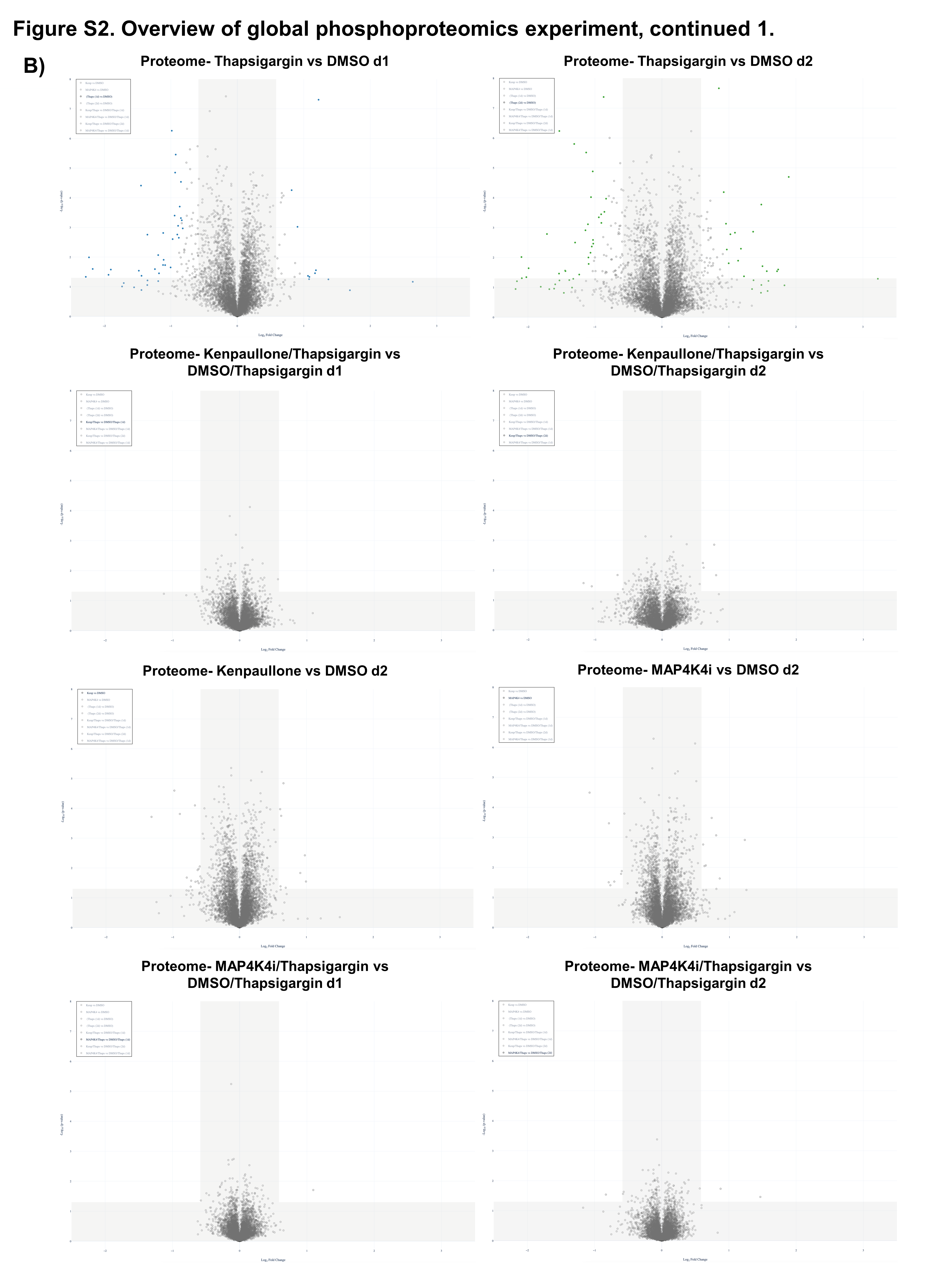

Supplement: Supplementary file 8 [file Image_3.TIF]

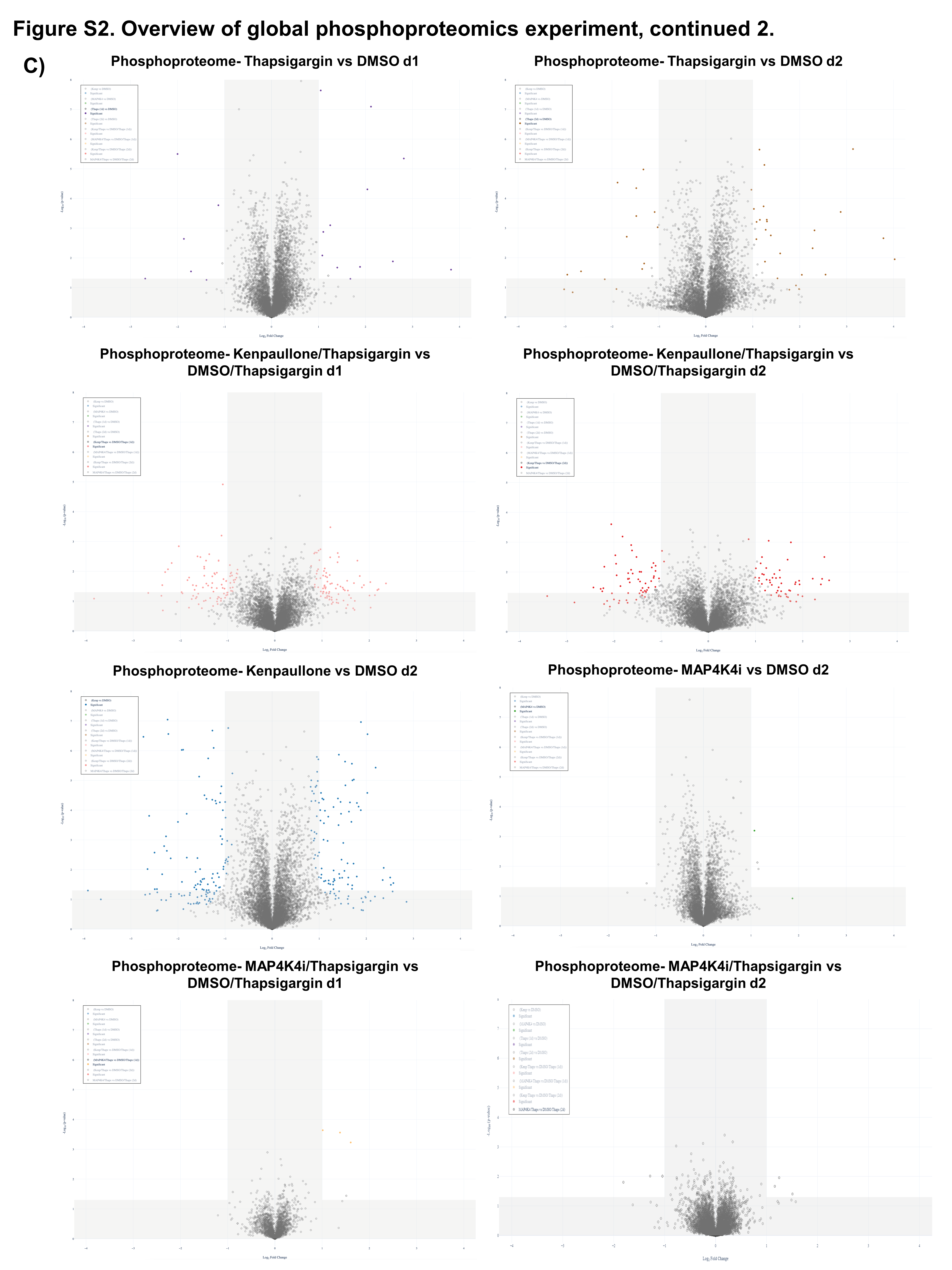

Supplement: Supplementary file 9 [file Image_4.TIF]

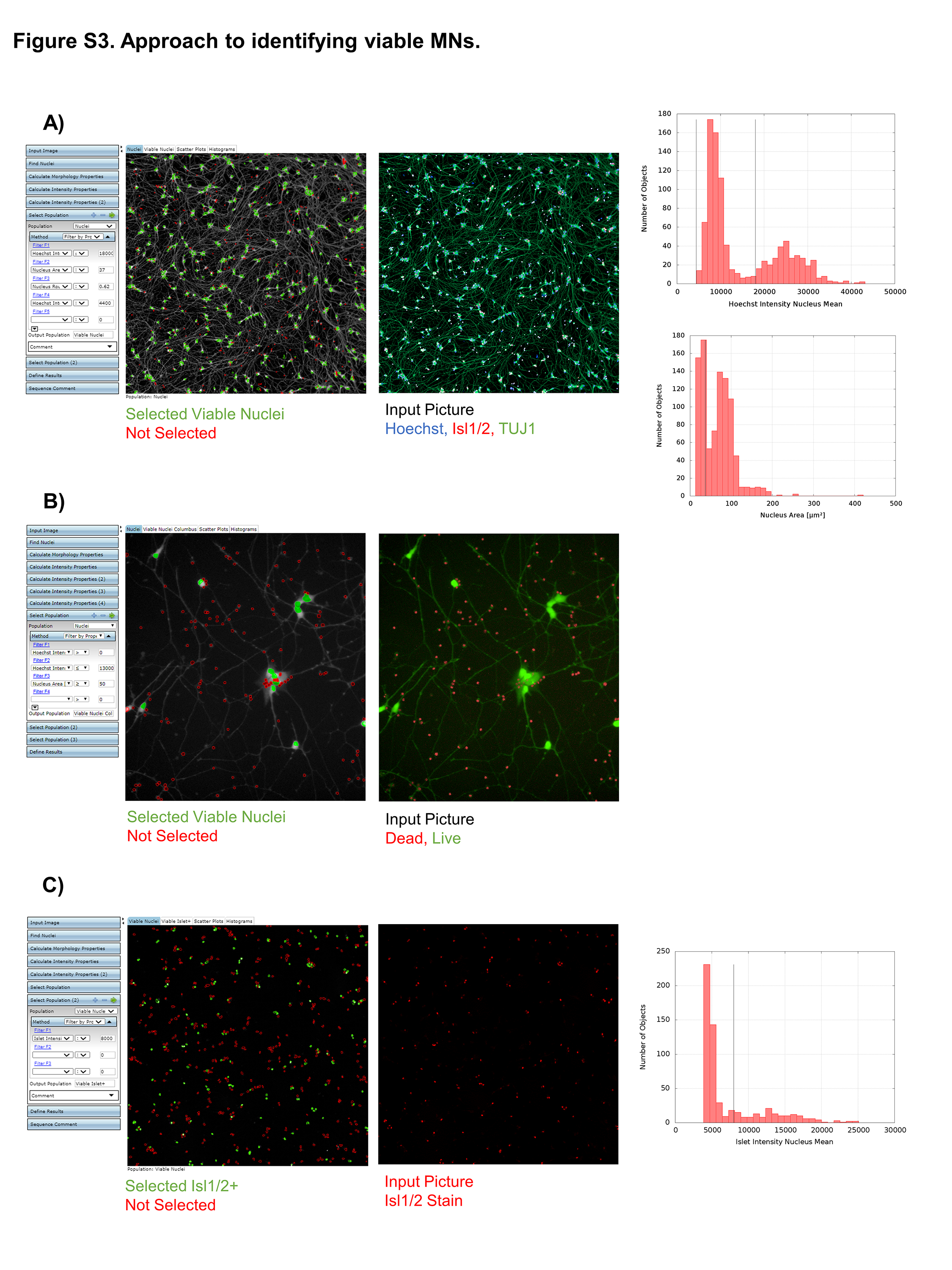

Supplement: Supplementary file 10 [file Image_5.TIF]

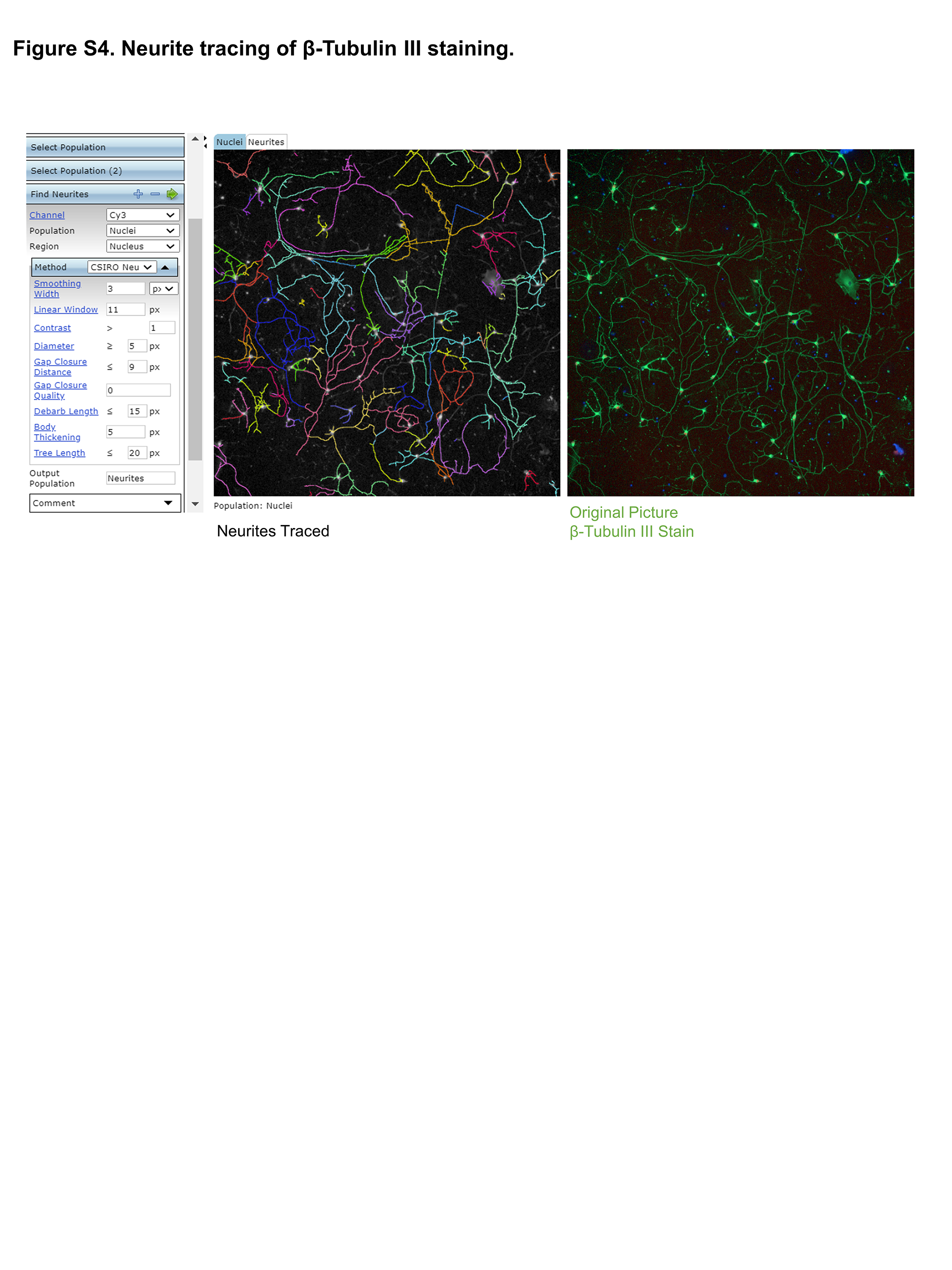

Supplement: Supplementary file 11 [file Image_6.TIF]
